# Possible clinical implications and future directions of managing bacterial biofilms in cutaneous leishmaniasis wounds

**DOI:** 10.1186/s41182-022-00455-y

**Published:** 2022-08-26

**Authors:** T. D. Jayasena Kaluarachchi, Paul M. Campbell, Renu Wickremasinghe, Shalindra Ranasinghe, Surangi Yasewardene, Hiromel De Silva, Andrew J. McBain, Manjula Weerasekera

**Affiliations:** 1 Department of Parasitology, Faculty of Medical Sciences, University of Sri Jayewardenepura, Nugegoda, Sri Lanka; 2 Division of Pharmacy and Optometry, School of Health Sciences, Faculty of Biology, Medicine and Health, The University of Manchester, Manchester, UK; 3 Department of Anatomy, Faculty of Medical Sciences, University of Sri Jayewardenepura, Nugegoda, Sri Lanka; 4 Dermatology Clinic, Base Hospital, Tangalle, Sri Lanka; 5 Department of Microbiology, Faculty of Medical Sciences, University of Sri Jayewardenepura, Nugegoda, Sri Lanka; 6 Sri Lanka Institute of Biotechnology (SLIBTEC), Pitipana, Homagama, Sri Lanka

**Keywords:** Cutaneous leishmaniasis, Management, Antibiotics, Debridement, Biofilms, Microbiome

## Abstract

Cutaneous leishmaniasis (CL) lesions are chronic and result in disfiguring scars. The microbiological aspects of these wounds have not been systematically investigated. We have recently reported that 61.5% of CL wounds in a Sri Lankan cohort harboured bacterial biofilms, mainly composed of bacilli, *Enterobacteriaceae*, and Pseudomonas, which could delay wound healing. We have additionally reported that biofilms were significantly associated patients over 40 years of age, discharge, pain and/or itching of the wound, and high pus cell counts. Using this as background knowledge and other relevant literature, we highlight the importance of investigating the role of biofilms in CL wound healing, clinical indicators, cost-effective laboratory tests involving less invasive sampling techniques for diagnosing biofilms and potential therapeutic options for biofilm-containing CL wounds, such as adjunctive application of wound debridement and antimicrobial treatment along with anti-parasitic drugs.

## Background

Cutaneous leishmaniasis (CL) is the commonest clinical manifestation of leishmaniasis, a neglected tropical disease [1]. Even though not life-threatening CL causes disfiguring scars leading to social stigma [2]. Cutaneous leishmaniasis is endemic in more than 70 countries, mostly affecting economically poor populations.

Studying CL wound microbiology is important for effective case management. It is known that patients with ulcerated skin lesions are prone to develop secondary-bacterial infections and, biofilms [3]. Wound biofilms are frequently polymicrobial, pathogenic, have specific clinical and therapeutic implications like reduced antimicrobial susceptibility, enhancing inflammation, and impeding fibroblast deposition. In CL wounds, the incidence of secondary-bacterial infection ranges between 20 and 81% [4, 5]. However, minimal evidence is available concerning CL wound biofilm formation.

**Fig. 1:**
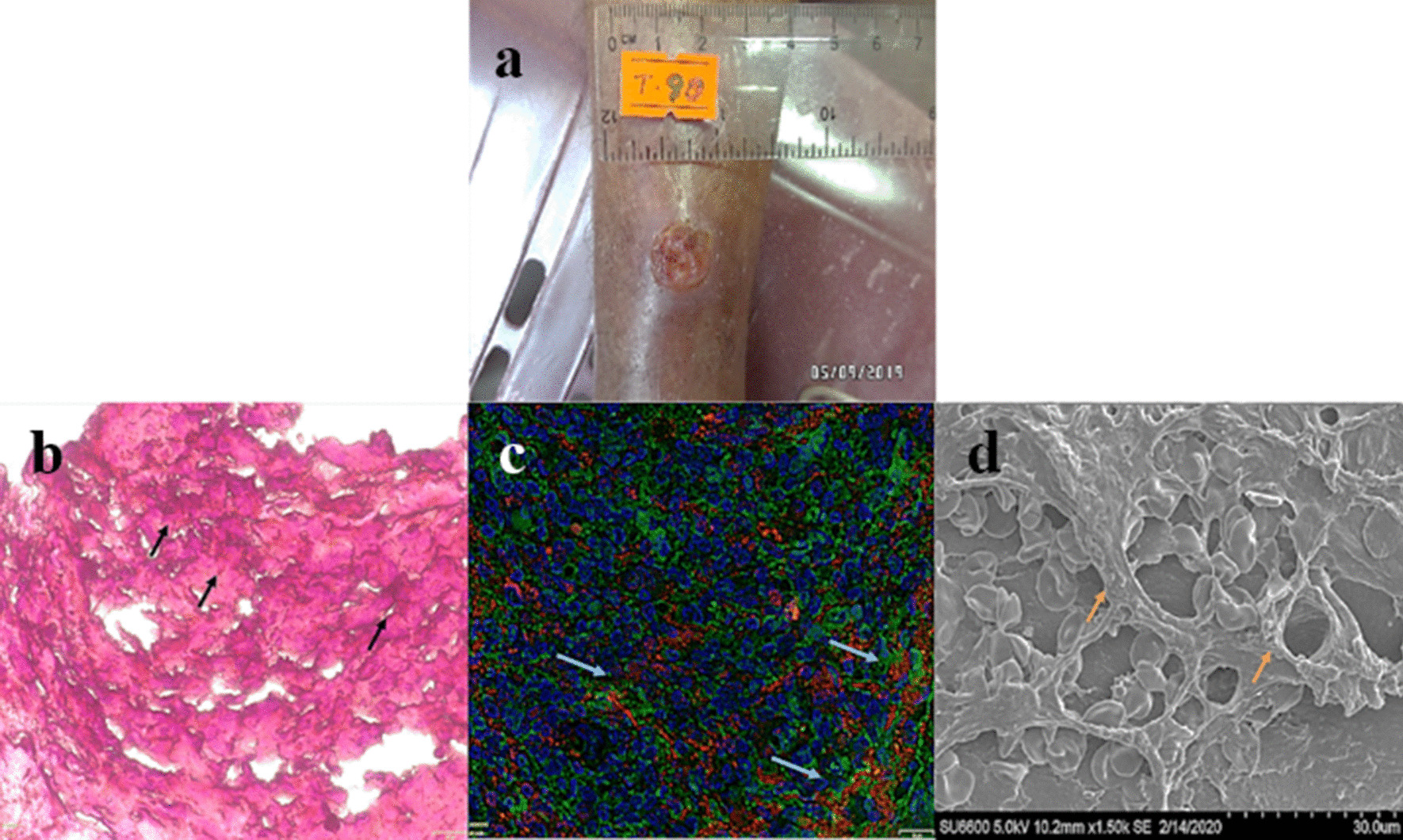
Visualization of bacterial biofilms in cutaneous leishmaniasis wound. Wet ulcer (**a**); Gram staining, the extra-polymeric substances (EPS) is stained in pinkish orange with the Safranin dye (**b**); fluorescence in situ hybridization—bacteria in red due to Cyanine 3-tagged Eu-bacterial rRNA probe, EPS in green due to Concanavalin A-conjugated Alexa Fluor 488 and tissue nuclei in blue due to DAPI staining (**c**), scanning electron microscopic image (**d**). Arrows indicate the biofilm

We have recently investigated biofilm formation in CL wounds [6]. In this study, 39 ulcerated CL wounds, collected over 1 year (2019–2020) from an endemic area of Sri Lanka, were subjected to Gram staining, fluorescent in situ hybridization (FISH) and scanning electron microscopy (SEM) imaging, to visualize bacterial biofilms (Fig. 1). Further, we described their clinico-demographic associations and the composition of the biofilms by Illumina MiSeq sequencing with V3–V4 region amplification.

We found that 61.5% (24/39) of the local ulcerated CL wounds harboured bacterial biofilms (as detected by FISH). These biofilms were of ~ 7 µm to 140 µm in size. SEM had a similar performance to FISH in visualizing biofilms (59% biofilm-positives). However, Gram stain only detected 35.9% of these biofilms. The biofilms were significantly associated with wounds with symptoms (pain and itching, 12/13), discharge (19/23), high pus cell counts (>25 pus cells/low power field, 9/9) and age of >40 years (20/27). Also, even though not statistically significant more biofilm formation was observed in wounds < 3 months of duration (17/23) and wounds with high parasite loads (> 1–10 amastigotes/100 microscopic fields, 15/22). A significant proportion of the clinically non-infected wounds (13/25; redness, warmth, swelling, and fever were considered to confirm clinical infection) had biofilms. The biofilm-positive wounds had significant lower community evenness compared to biofilm-negative wounds and were dominated by OTUs belonging to class *bacilli*, family *Enterobacteriaceae*, and genus *Pseudomonas*.

In this short report, we highlight the importance of investigating the role of biofilms in CL wound healing, their clinical indicators, cost-effective laboratory tests involving less/non-invasive sampling techniques for diagnosing biofilms and potential therapeutic options for these biofilm-containing CL wounds, such as adjunctive application of wound debridement and antimicrobial treatment along with anti-parasitic drugs. The discussion is partly based on our previous findings [6] but more broadly considers recent literature on chronic wound biofilms. We hope that this will introduce the concept of biofilm-specific CL wound management.

## Main text

Biofilms in chronic wounds are responsible for delayed wound healing [7]. There poses a dilemma regarding CL wounds. While the role of pathogenic bacteria and fungi in delaying the healing of CL wounds is understood [8], it has been claimed that infections with *Staphylococcus aureus, Pseudomonas aeruginosa, Enterococcus faecalis, Streptococcus pyogenes* and *Candida parapsilosis* has no impact on the epithelialization and healing time in CL wounds [5]. A Sri Lankan study on biofilms in diabetic wounds reported that most chronic diabetic foot wounds are colonized with *Pseudomonas* spp. [9]. Under similar settings, we found that the biofilms of ulcerated local CL wounds were mainly composed by class *bacilli*, family *Enterobacteriaceae*, and genus *Pseudomonas* [6]*. Pseudomonas* is one of the most common biofilm-forming wound pathogens isolated from chronic wounds. Evidence shows that early diagnosing of *Pseudomonas* in wounds and prompt treatment will minimize most of the undesirable wound outcomes [10]. Staphylococci are known to be highly efficient in biofilm making and impair wound healing [11]. Planktonic streptococci form into well-developed, antibiotic-resistant biofilms within 6–12 h [12]*.* Also, *Enterobacter* spp. significantly associated with poor wound healing [13]*.* With such a background, it would be interesting to see how the bacterial biofilms in CL wound affect their healing. This could be evaluated by a longitudinal study with complete wound healing as an endpoint.

Further, the CL wound microenvironment may play an important role in the formation of these bacterial biofilms, i.e. the observed early formation of the biofilms (< 3 months) [6] could be due to the acidic pH found in CL wounds. Acidic pH levels have been found to facilitate biofilm formation in vitro [14] and further identified as a factor enhancing antibiotic resistance in biofilms formed by *Pseudomonas aeruginosa* [15]. Vice versa, biofilms could affect the CL wound microenvironment as well. It has been found that the altered bacterial burden can change the immune microenvironment of CL wounds by recruiting more neutrophils, IL-1β and activation of IL-17A [16]. Investigating these changes would be important to open new paths in the management of CL wounds.

Another interesting fact to look at would be the clinical indicators of CL wound biofilms. Clinical assessment on the presence of biofilms in wounds is challenging. The presence of warmth, redness, swelling and fever would suggest an ongoing infection at the wound site [17]. However, these features would not suggest the definite presence of a biofilm. Cutaneous leishmaniasis wounds are mostly asymptomatic unless super-infected. The significant association of age of > 40 years, discharge, pain and/or itching in CL wounds [6] could be considered as possible clinical indicators of biofilms in CL wounds and need to be further evaluated. This will facilitate clinical judgement and prompt treatment could be started.

Laboratory confirmation of biofilms by FISH/SEM needs invasive sampling and sophisticated infrastructure. In contrast to a pilot study conducted on diabetic wounds [18], local experience has proven that Gram staining is less accurate in biofilm detection [6, 9]. FISH, SEM and Gram staining, all use biopsy tissues to confirm biofilms. Invasive sampling can result in iatrogenic infection. Thus, more investigations should be carried out to find a cost-effective test for biofilm confirmation, and minimally/non-invasive mode of sampling, i.e. point-of-care fluorescence imaging of wounds which has been introduced as a successful method of identifying possible *Pseudomonas* infections [10].

Protozoans in the environment have a predator/grazing effect against bacterial biofilms. The bacterivorous nature of the *Leishmania* parasite has not been investigated. However, in our study, we noted many wounds with high parasite loads had bacterial biofilms [6]. This raises the possibility that the local strain *Leishmania donovani* has less predatory qualities against bacterial biofilms and needs further investigations.

The size of the CL wound biofilms (7–140 µm) [6] was lower than what has been reported with diabetic foot wounds (12–400 µm) [9]. This could probably be due to early sampling of the reported CL wounds [6] or the patchy distribution of biofilms. In the case of the latter, it would be beneficial to explore the spread of the biofilms and to see how the composition of the organisms differs across the CL wound bed. The best method to do this is by using FISH assay with differently tagged species-specific probes. This will facilitate the possible use of a targeted antibiotic treatment adjunctive to anti-parasitic drugs to improve the healing of these wounds.

However, there is a lack of agreement about the effect of antibiotics on CL wounds [6]. Since the Sri Lankan CL wounds were dominated by biofilms formed by class *bacilli*, family *Enterobacteriaceae*, and genus *Pseudomonas* [6], further investigations are needed to evaluate an antibiotic effective against all the three groups of the above organisms, i.e. cephalosporin which is commonly used for skin and soft tissue infections [19].

Regardless of this, biofilms are typically highly tolerant to antibiotic treatment [20]. Wound debridement is another method of successfully treating the biofilm harbouring chronic wounds [21]. Wound debridement could be tailored according to the characteristics of the identified biofilm, i.e. debridement could be coupled with antibiotics targeting the specific group of bacteria composing the biofilm [21]. The International Wound Bed Preparation Advisory Board has recommended a four-step algorithmic approach to manage infected wounds [22]. The steps include (1) debridement of tissue, (2) management of infection/inflammation, (3) balancing moisture by appropriate dressings, and (4) wound edge assessment [22]. This application has been experimented with chronic wounds and has been observed to be advantageous in terms of wound healing and cosmetic outcome [23]. Therefore, it is important to conduct more investigations to find out how applicable this procedure is concerning CL wounds with biofilms.

## Conclusion

Like any other chronic wound, microbiology likely plays an important role in wound healing/management of CL wounds. We encourage more research directed towards investigating the role of bacterial biofilms in CL wounds, their clinical indicators, the efficacy of anti-biofilm treatment modalities including wound debridement and topical antimicrobials, along with the standard of care, which includes the use of systemic and local anti-parasitic drugs for the treatment of this condition. This will impact management policy-making and treatment regarding CL wounds and benefit many who suffer from the chronicity of these wounds.

## Data Availability

Not applicable.

## Abbreviations

CL: Cutaneous leishmaniasis
EPS: Extra-polymeric substances
EUB: Eu-bacterial
FISH: Fluorescence in situ hybridization assay
IM: Intramuscular
IV: Intravenous
